# Employing the Next Generation: Isn’t it time to Make the Transition

**DOI:** 10.5812/traumamon.12873

**Published:** 2013-08-11

**Authors:** Shahram Nazerani

**Affiliations:** 1 Firuzgar Medical Center, Iran University of Medical Sciences, Tehran, IR Iran

**Keywords:** Hospital Administration, Private Sector, Healthcare Sector

During a general assembly session in a prestigious private hospital, I watched with fascination as all the nominees for the board of directors were above 65 years of age; some were even in their eighties. The homage and tribute to the seniors was so great that the younger doctors were too embarrassed to nominate themselves while the “seniors” competed most fervently for candidacy (1). Statistics show that these senior candidates are not actively involved anymore and it puts the burden of day-to-day hospital activities on the shoulders of younger doctors(2). Isn't it time that the board of directors which make the administrative decisions of the hospital to include the younger generation as well as the seniors? In western countries the medical licensure and recertification programs, intolerable private practice overheads, the fierce competition and malpractice suits of the senior doctors are the regulating mechanisms which compel a senior doctor to retire. On average the retirement age is 65 for surgeons and 68 for internists. Altogether there is an age limit which the doctor should consider retiring by, so the private as well as government hospital workforce are always renewed by the younger generations filling the shoes of the retired doctors. In our country the government hospitals have a retirement program for doctors but the inadequacy of the retirement package forces them to shift their practice to a private hospital or office where they can work as long as they want. The newly established private hospitals in our country are run mostly by the younger generations and while we see most are flourishing, older private hospitals with senior old fashioned management systems are either going bankrupt and closing due to financial or managerial problems or are lagging far behind the new ones in terms of hospital bed occupancy, number of surgical procedures and implementation of the new modalities of treatment. I think that the older private hospitals must accept the presence of the younger generation in the board of directors not only for the young generation to gain experience but also to trigger the much needed contemporary changes in management. The other, albeit unpleasant, solution is to implement a mandatory retirement age in the private sector otherwise the hospital may face the possibility of bankruptcy.
